# High-Titer Anti-ZSCAN1 Antibodies in a Toddler Clinically Diagnosed with Apparent Rapid-Onset Obesity with Hypothalamic Dysfunction, Hypoventilation, and Autonomic Dysregulation Syndrome

**DOI:** 10.3390/ijms25052820

**Published:** 2024-02-29

**Authors:** Vlad Tocan, Akari Nakamura-Utsunomiya, Yuri Sonoda, Wakato Matsuoka, Soichi Mizuguchi, Yuichiro Muto, Takaaki Hijioka, Masao Nogami, Daiki Sasaoka, Fusa Nagamatsu, Utako Oba, Naonori Kawakubo, Hiroshi Hamada, Yuichi Mushimoto, Pin Fee Chong, Noriyuki Kaku, Yuhki Koga, Yasunari Sakai, Yoshinao Oda, Tatsuro Tajiri, Shouichi Ohga

**Affiliations:** 1 Department of Pediatrics, Graduate School of Medical Sciences, Kyushu University, Fukuoka 812-8582, Japan; 2 Department of Genetic Medicine/Pediatrics, Graduate School of Biomedical and Health Sciences, Hiroshima University, Hiroshima 734-8511, Japan; 3 Department of Pediatrics, Hiroshima City North Medical Center Asa Citizens Hospital, Hiroshima 731-0293, Japan; 4 Division of Neonatal Screening, National Center for Child Health and Development, Tokyo 157-8535, Japan; 5 Emergency and Critical Care Center, Kyushu University Hospital, Fukuoka 812-8582, Japan; 6 Department of Pediatrics, Japanese Red Cross Kumamoto Hospital, Kumamoto 861-8520, Japan; 7 Department of Pediatrics, Graduate School of Medical Sciences, Kumamoto University, Kumamoto 860-8556, Japan; 8 Department of Pediatric Surgery, Graduate School of Medical Sciences, Kyushu University, Fukuoka 812-8582, Japan; 9 Department of Anatomic Pathology, Graduate School of Medical Sciences, Kyushu University, Fukuoka 812-8582, Japan

**Keywords:** ROHHAD syndrome, anti-ZSCAN1 antibodies, autoimmune encephalitis, paraneoplastic syndrome, ganglioneuroblastoma, severe obesity, hypoventilation

## Abstract

Severe obesity in young children prompts for a differential diagnosis that includes syndromic conditions. Rapid-Onset Obesity with Hypothalamic Dysfunction, Hypoventilation, and Autonomic Dysregulation (ROHHAD) syndrome is a potentially fatal disorder characterized by rapid-onset obesity associated with hypoventilation, neural crest tumors, and endocrine and behavioral abnormalities. The etiology of ROHHAD syndrome remains to be established, but recent research has been focusing on autoimmunity. We report on a 2-year-old girl with rapid-onset obesity during the first year of life who progressed to hypoventilation and encephalitis in less than four months since the start of accelerated weight gain. The patient had a high titer of anti-ZSCAN1 antibodies (348; reference range < 40), and the increased values did not decline after acute phase treatment. Other encephalitis-related antibodies, such as the anti-NDMA antibody, were not detected. The rapid progression from obesity onset to central hypoventilation with encephalitis warns about the severe consequences of early-onset ROHHAD syndrome. These data indicate that serial measurements of anti-ZSCAN1 antibodies might be useful for the diagnosis and estimation of disease severity. Further research is needed to determine whether it can predict the clinical course of ROHHAD syndrome and whether there is any difference in antibody production between patients with and without tumors.

## 1. Introduction

Obesity in children is a global issue. The raising pace of severe obesity appears to have attenuated at the beginning of the 21st century [1], but there are concerns that it started to increase again in the younger population during the SARS-CoV-2 pandemic [2,3,4]. Some investigators reported an inverse correlation between age and body mass index (BMI) in patients hospitalized for COVID-19 [5]. Although the percentage of obese toddlers is low compared to older children and adults, obesity in infants and children can be a risk factor for later obesity and development of metabolic syndrome [6,7].

Extreme obesity in young children prompts for the differential diagnosis of syndromic (genetic, nongenetic, and endocrine) disorders [8], including Prader–Willi syndrome, Bardet–Biedl syndrome, leptin/leptin receptor deficiency, and others. Rapid-Onset Obesity with Hypothalamic Dysfunction, Hypoventilation, and Autonomic Dysregulation (ROHHAD) syndrome is a rare pathological entity that was proposed in 2007 to define a group of patients with late-onset central hypoventilation, accompanied by a number of symptoms, including rapidly progressing obesity, water imbalance, endocrine abnormalities, autonomic dysfunction, and strabismus [9]. Sporadic cases have been reported since 1965, indicating a condition different from congenital central hypoventilation syndrome [10,11]. The addition of ‘neural crest tumors’ (NET) to the nomenclature, ROHHAD-NET, was proposed by Bougnères et al. [12] in 2008 because these tumors are observed in 50–60% of patients [12,13]. Until recently, only about 50 individual patients have been described in the medical literature, and data from around 150 patients have been analyzed in case series and reviews [13,14], indicating that ROHHAD syndrome is a very rare but presumably underdiagnosed disease. This syndrome leads to life-threatening hypoventilation, with a reported mortality rate of approximately 20% [13,14]. Efforts to identify a unique cause have not been met with success until now, although an autoimmune mechanism hypothesis appears promising [15,16].

Here, we report on a 2-year-old girl with rapid-onset obesity during the first year of life who progressed to hypoventilation and impaired consciousness in less than four months since the start of weight gain. Her symptoms and treatment course suggested a clinical diagnosis of ROHHAD syndrome. The patient had a high serum anti-ZSCAN1 antibody titer that did not decline after acute phase treatment.

## 2. Methods

### 2.1. Assays for Autoimmune Encephalitis Antibodies

All cerebrospinal fluid (CSF) and serum samples were frozen immediately after collection at −30 °C until analysis. We tested acute serum samples for MOG positivity using a fixed immunofluorescence cell-based assay (CBA; Euroimmun, Germany) by visual observation of the fluorescein-labelled binding MOG-IgG [17]. The CSF sample was tested for autoantibodies against NMDA, CASPR2, AMPA1/2, LGI1, DPPX, and GABA-RB1/2 with a similar cell-based assay (CBA; Euroimmun, Germany) [18].

### 2.2. Assays for ZSCAN1 and Na_x_ Antibodies

The antibody response to ZSCAN1 and Na_x_ was analyzed by ELISA as previously described [19,20]. The plates were incubated with the N-terminal GST-tagged recombinant human ZSCAN1 protein (Q8NBB4-1) or SCN7A protein (HQ258196.1), which were expressed using a wheat germ cell-free system, for 1 h at 25 °C. Human IgG, which was expressed using a wheat germ cell-free system, was used as the positive control, and mock (H_2_O instead of messenger RNA) was used as the negative control. The plates were incubated with serum samples for 1 h at 25 °C. Next, the wells were incubated with goat antihuman IgG (1:10,000 dilution; cross-adsorbed secondary horseradish peroxidase-conjugated antibody [H + L]; A18811, Thermo Fisher Scientific, Waltham, MA, USA) for 1 h at 25 °C, followed by incubation with 100 μL tetramethylbenzidine–H_2_O_2_ (Pierce TMB substrate kit, ThermoFisher Scientific, Waltham, MA, USA). After 30 min of incubation at 25 °C, the reaction was stopped by the addition of 100 μL of 2 M H_2_SO_4_, and the intensity of the yellow dye was measured at 450 nm. A calculated titer above 40 was considered a positive response.

### 2.3. Case Presentation

**Figure 1:**
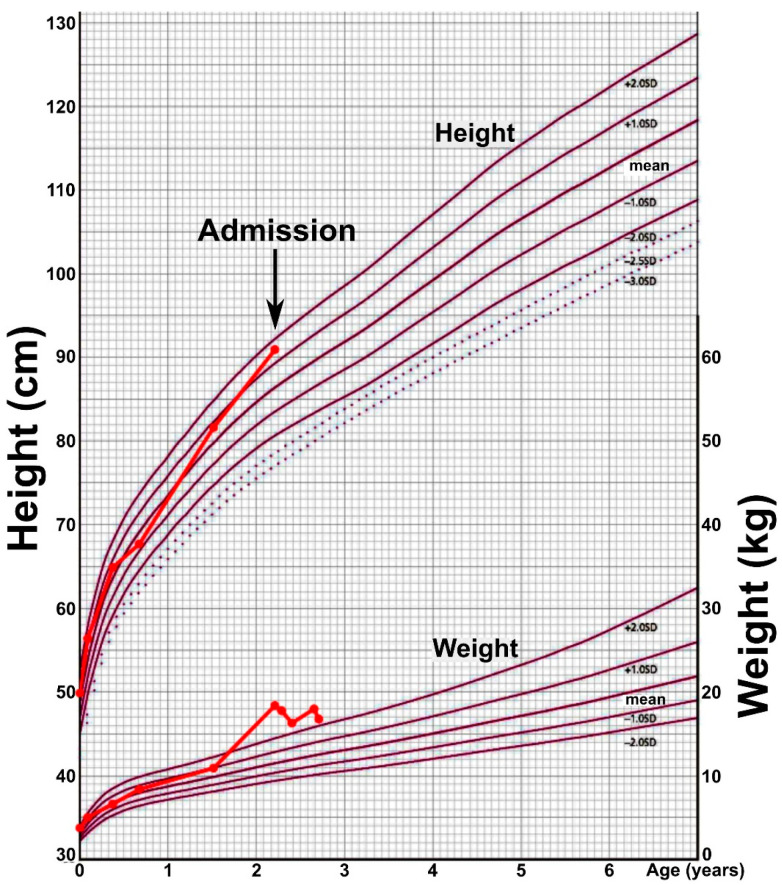
Growth chart (chart for 0–6-year-old Japanese girls provided by the Japanese Society of Pediatric Endocrinology and based on values reported in [21]).

**Figure 2:**
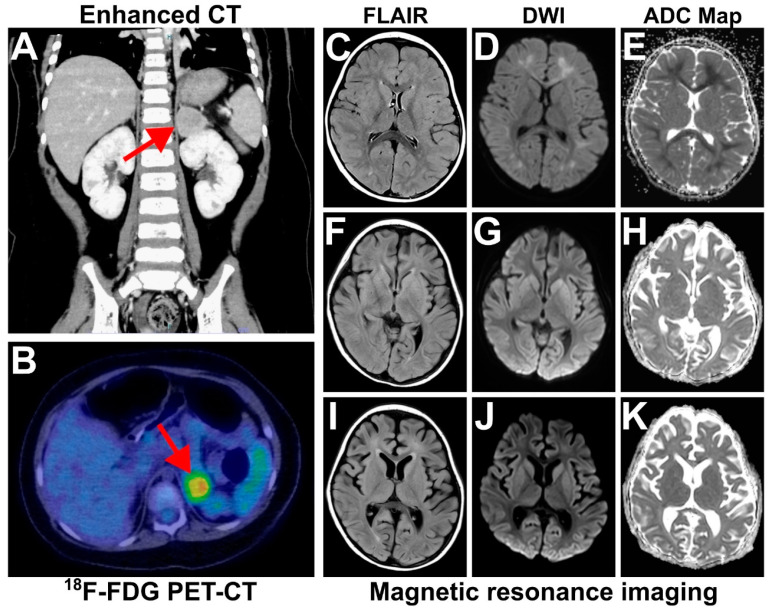
Main radiologic findings. (**A**) Enhanced computer tomography (CT) scan: coronal reconstruction. The arrow indicates the left adrenal tumor. (**B**) ^18^F-FDG positron transmission tomography–computer tomography (PET-CT) scan: axial slice. The arrow indicates high tracer uptake (SUVmax 4.69) in the left adrenal tumor. (**C**–**K**) Magnetic resonance images of the patient’s brain: (**C**–**E**) Day 1 (taken at the previous hospital), (**F**–**H**) Day 15 (before treatment), (**I**–**K**) Day 37 (after treatment).

A 2-year-old girl with rapid-onset obesity was admitted to the pediatric intensive care unit because of respiratory failure and a left adrenal tumor. The patient was born after an uneventful pregnancy at a gestational age of 39 weeks and 2 days, with a birth weight of 3894 g (+2.88 SD) and a length of 50.0 cm (+0.56 SD), without asphyxia. This healthy newborn had no family history of inheritable diseases. Her mother’s and father’s BMIs were 25.2 kg/m^2^ and 28.0 kg/m^2^, respectively. During newborn screening for congenital endocrine and metabolic diseases, congenital hypothyroidism was initially suspected but finally excluded. Up to 1 year and 6 months, the patient had normal physical and neurological development, and, at that time, her weight was 11.1 kg (+1.16 SD; BMI-SDS +0.75 SD), as measured at an official medical check-up. According to her parents, her body weight was around 12 kg (+0.97 SD) at 1 year and 11 months, and she was a rather shy child, often crying when in pain or when she was scolded and was picky with food. Shortly before 24 months of age, the patient’s pickiness suddenly disappeared. She began to eat all types of food in increasing amounts, gaining weight rapidly (Figure 1). She presented with strabismus over the next two months and stopped crying in any kind of uncomfortable situations, indicating a possible change in character. She also started to snore during sleep, and her parents noticed that her breathing was very shallow, and it sometimes stopped briefly. At 2 years and 2 months, she developed cold-like symptoms (fever, nasal discharge, and cough). Three days later, she was found unresponsive and was taken to the emergency room of a neighborhood hospital. Upon arrival, the Glasgow Coma Scale score was 3 points (E1V1M1), and the patient’s blood CO_2_ pressure was 96 mmHg, indicating hypercapnia. After tracheal intubation, the blood CO_2_ pressure decreased to 43 mmHg. Tests for influenza and COVID-19 viruses were negative. An electroencephalogram indicated high amplitude slow waves. Empirical treatment for encephalitis with antibiotics, antiviral drugs, and mitochondrial therapy was started. During the next three days, the patient developed hypernatremia (162 mmol/L), and an abdominal enhanced computer tomography showed a weakly enhanced, lobulated 30 mm mass with a distinct boundary in the left adrenal gland as well as the enlargement of an adjacent lymph node (Figure 2A). On Day 4 of admission, she was transferred to our hospital for further investigations and treatment.

**Figure 3:**
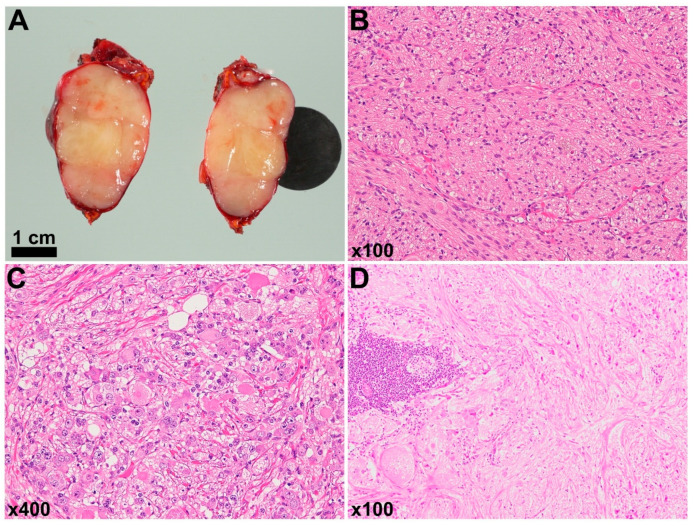
Tumor pathology. (**A**) Macroscopic image of the resected left adrenal tumor. (**B**,**C**) Hematoxylin–eosin-stained images of tumor tissue showing proliferation of short spindle-shaped cells in a fascicular pattern as well as focal calcifications and aggregates of differentiating neuroblasts with abundant cytoplasm and blastic small round cells with neuropil background, indicating ganglioneuroblastoma intermixed. (**D**) Hematoxylin–eosin-stained image of the resected adjacent lymph node showing metastasis of neuroblastic cells.

At admission, her weight was 18.5 kg (+3.93 SD), and her height was 91.0 cm (+1.76 SD), with a BMI of 22.34 kg/m^2^ (+4.52 SD) (Figure 1). She was already intubated and under sedation. The patient was obese but without evident signs of central obesity. The main laboratory tests (Table 1) indicated hypernatremia, hyperprolactemia, low IGF-1, and slightly elevated NSE. CSF was characterized by high sodium values and the presence of oligoclonal bands without pleocytosis (cell count 3/μL) (Table 1). A multiplex PCR panel (Biofire Diagnostics, Salt Lake City, UT, USA) for 14 common encephalitis-causative pathogens performed on the CSF sample was negative. Magnetic resonance imaging (MRI) of the brain performed at the previous hospital on Day 1 showed regions of deep white matter with a high intensity signal on diffusion-weighted images (DWI) and with low apparent diffusion coefficient (ADC) values (Figure 2C–E). Based on the history of rapidly progressing obesity, central hypoventilation, hypothalamic abnormalities (hypernatremia and hyperprolactemia), autonomic dysfunction (strabismus and possibly elevated pain threshold), and the presence of an adrenal tumor, a diagnosis of ROHHAD-NET syndrome was considered. The left adrenal mass and the adjacent lymph node had high ^18^F-FDG uptake on a positron emission tomography (Figure 2B). Although there was no strong indication that the mass was a functional tumor because the patient’s consciousness state did not improve and mild global cerebral atrophy appeared with new high intensity signals in the bilateral caudal nuclei on Day 15 (Figure 2F–H), we could not exclude the possibility that the patient’s encephalopathy was the presentation of a paraneoplastic syndrome. The tumor was surgically resected on Day 18 and pathologically diagnosed as ganglioneuroblastoma intermixed with metastasis to one adjacent lymph node (Figure 3). After daily immunoglobulin infusions from Day 23 to Day 27, the patient’s consciousness level improved, and the high-intensity signal on MRI regressed (Figure 2I–K). Tests for serum antibodies against Na_x_ and MOG and for CSF antibodies against NMDA, CASPR2, LGI1, AMPA1/2, GABA-RB1/2, and DPPX, all known to be related to autoimmune encephalopathy, were negative. We also tested for the serum anti-ZSCAN1 antibody, a recently reported possible marker for ROHHAD syndrome [15,20], and found a remarkably high titer that did not decrease after acute phase treatment (Table 2).

On Day 22, the patient was extubated and placed on noninvasive positive pressure ventilation. Her condition improved to the level that she could obey instructions given by the medical staff and watch cartoons during the day, but she was unable to sit independently or speak. Sleep apnea studies performed after extubation indicated that the patient was still hypoventilating, with high PaCO_2_ levels (70–90 mmHg) during sleep. As noninvasive ventilation was not efficient, a tracheostomy was performed on Day 32, after which PaCO_2_ decreased to 50–60 mmHg. The patient was transferred back to the previous hospital on Day 46, then retransferred to a regional university hospital, where she was treated for another three months before being released. At six months after the acute neurological onset, the condition of the patient was stable, with a weight of 16.9 kg (+2.88 SD) and a BMI of 21.34 kg/m^2^ (+3.84 SD). At the age of 2 years and 8 months, she can walk and speak during the day, but she still requires pressure support ventilation during sleep, and her latest venous CO_2_ pressure was 48 mmHg. Her serum sodium levels are around 136 mmol/L, and her endocrine abnormalities (low IGF-1 and hyperprolactinemia) are unchanged.

## 3. Discussion

The female toddler in our report had all the known hallmarks of ROHHAD syndrome: rapidly progressing obesity, late-onset central hypoventilation, a neural crest tumor, hypernatremia, hyperprolactinemia, autonomic dysregulation, neurobehavioral changes, and strabismus. Compared to the statistics of 46 ROHHAD syndrome patients described by Harvengt et al. [13], our patient started to gain weight at 1.9 years (median 3.1 years), had behavioral changes around 2 years (median 3.8 years), and was diagnosed with central hypoventilation and ganglioneuroblastoma at 2.2 years (median 5.3 years and 4.75 years, respectively), indicating that, among reported patients up to the present, she had a very early onset and very rapid progression of the symptoms. The progression in an extremely short time (less than 4 months) from obesity onset to symptomatic hypoventilation in our patient underlines the severity of this rare syndrome. Our patient was overweight (+2.88 SD) at birth, but by 6 months of age, her weight entered the normal range and remained in range at least until 1.5 years (Figure 1). The weight curve clearly shows that obesity developed rapidly around the age of 2 years, coinciding with the sudden start of hyperphagia, as reported by her parents. It is not evident whether high birth weight is related to obesity in ROHHAD syndrome. Although the apparent height increase after the onset of weight gain in this patient is not considered typical for ROHHAD syndrome, in which growth is usually attenuated. The follow-up period has been too short to evaluate height, albeit the patient’s persistently low IGF-1 values might be a sign of future growth attenuation.

The patient had an adrenal ganglioneuroblastoma intermixed at diagnosis. In over half of ROHHAD syndrome patients, neural crest tumors, mostly ganglioneuroma or ganglioneuroblastoma, are identified at the time of diagnosis or during follow-up [12,13]. Neural crest tumors include neuroblastoma/ganglioneuroblastoma nodular, ganglioneuroblastoma intermixed, ganglioneuroma maturing, and ganglioneuroma mature, and they are considered part of a spectrum of maturing tumors, with a diagnosis age increasing in the above order (median diagnosis age of 9, 61, 71, 125 months, respectively), as reported in large cohorts [23,24]. The median diagnosis age of ganglioneuroblastoma (4.75 years) in ROHHAD syndrome [13] is similar to the above data, indicating a certain correlation between tumor development and ROHHAD syndrome progression. Although the presence of neural crest tumors is one of the main features of the syndrome, to the best of our knowledge, large cohorts of these tumors do not mention ROHHAD syndrome or its resembling features, albeit some authors report the association of neural crest tumors with opsoclonus myoclonus ataxia, another paraneoplastic syndrome, or type 1 neurofibromatosis [24,25,26,27]. 

Due to the prolongation of encephalitis symptoms in our patient, we could not exclude a paraneoplastic syndrome caused by the patient’s tumor. The possibility of ROHHAD syndrome manifesting as a paraneoplastic syndrome has been discussed extensively, but no decisive conclusion has been reached because tumors are found only in half of the patients, and the symptoms of ROHHAD syndrome appear to persist even after tumor resection [9,12,15,28]. Among diseases characterized by encephalitis as a paraneoplastic syndrome, anti-NMDAR encephalitis is a well-described autoimmune pathological entity, which has common symptoms, such as decreased levels of consciousness, autonomic instability, and hypoventilation, also reported in ROHHAD syndrome. Anti-NMDAR encephalitis manifests as a paraneoplastic syndrome in about half of the patients with anti-NMDAR seropositivity; 80% of patients who undergo tumor removal accompanied by first line immunotherapy (corticosteroids, intravenous immunoglobulin [IVIg], and/or plasma exchange) show substantial improvement [29]. These treatment options have also been used in patients with ROHHAD syndrome [16,28]. Our patient had a decreased level of consciousness, an increased FLAIR/DWI signal in the bilateral regions of deep white matter, and high amplitude slow waves on encephalogram, and these symptoms improved after tumor resection and IVIg infusion. The treatment of previously reported tumor patients usually includes both surgical resection and immunosuppressive therapy; therefore, it is not clear whether surgery alone is sufficient to alleviate symptoms of encephalitis as a ROHHAD syndrome comorbidity [13,28]. Although other etiologies for encephalitis in our patient were not completely excluded, the causal association with an infection or hypercapnia alone is less probable based on the lack of detection of any relevant pathogens or typical MRI findings.

Neuroblastoma, a tumor also originating from neural crest cells, has been reported to occur in association with congenital central hypoventilation syndrome (CCHS), a disorder that needs to be differentiated from ROHHAD syndrome. The etiopathogenesis of neurocristopathy syndrome (neuroblastoma, CCHS, and Hirschsprung disease) has been explained by the identification of *PHOX2B* variants in nearly all patients [30,31]. In addition, patients with CCHS usually show hypoventilation in infancy but no rapidly progressing obesity. Based on these differences, we considered the diagnosis of CCHS to be less probable in this patient and did not perform an analysis of *PHOX2B*. Furthermore, the rapidly progressing obesity, as well as the normal neurodevelopment before the advent of symptoms, did not suggest the diagnosis of other neurodevelopmental disorders, including Prader–Willi syndrome, Smith–Magenis syndrome, Wilms tumor, aniridia, genitourinary anomalies, and mental retardation (WAGR) syndrome [28].

Due to the young onset age, a genetic etiology of ROHHAD syndrome is suspected, but efforts to identify any causative genetic variants have been unsuccessful, even with whole genome analysis of patients and of their parents and/or twin siblings [32,33]. Candidate genes for ROHHAD syndrome have been reviewed comprehensively elsewhere [14,34]. A study using neurons derived from patients with ROHHAD syndrome, Prader–Willi syndrome, or CCHS found a larger variability in the gene expression in ROHHAD syndrome than in the other two syndromes [35]. In contrast, autoimmune etiology appears to be more plausible, especially in the context of the detection of autoantibodies and oligoclonal bands in the CSF of ROHHAD syndrome patients [36]. The present patient had CSF oligoclonal bands and high titers of serum anti-ZSCAN1 antibodies. The diagnostic utility of these antibodies and the treatment response to corticosteroids, IVIg, cyclophosphamide, or rituximab may support immune-based pathophysiology [34]. A few autopsy cases of ROHHAD syndrome also reported hypothalamic and pituitary inflammation with lymphocyte infiltration [37,38]. In this context, ROHHAD syndrome could be considered to have an autoimmune etiology, although further studies on other definitive cases are needed.

Patients with adipsic hypernatremia and endocrine abnormalities share a number of symptoms with ROHHAD syndrome patients but are not affected by hypoventilation. The serum of these patients has been found to include antibodies against the Na_x_ channel (sodium sensor) and the TPPV4 channel (osmotic pressure sensor) and to the subfornical organ (SFO) [16,39,40]. Anti-SFO antibodies have also been reported in some patients diagnosed with ROHHAD syndrome [19]. Recently, Mandel-Brehm et al. [15] identified autoantibodies to the ZSCAN1 protein in the CSF and sera of seven out of nine ROHHAD patients but in none of 125 controls. All seven patients had neuroblastic tumors, including four ganglioneuroblastomas. Based on the detection of anti-ZSCAN1 antibodies in tumor cells from a patient with neuroblastoma, they concluded that anti-ZSCAN1 antibodies could be a robust marker for diagnosing ROHHAD-NET. Our patient had a remarkably high titer of anti-ZSCAN1 antibodies that did not decrease after acute phase treatment. A previous report on anti-ZSCAN1 antibodies contains only qualitative histopathological assessments [15] that are not comparable with the present antibody titers. The mean anti-ZSCAN1 antibody titer in our preliminary analysis was 115 ± 85 (range 49–317) in a group of pediatric patients with ROHHAD and ROHHAD-NET [20]. Moreover, reported patients positive for anti-ZSCAN1 antibodies progressed to hypernatremia and hypoventilation [19,20]. We thus consider that the antibody titer may represent the severity and/or rapid progression of ROHHAD symptoms but that it did not directly affect the pathogenesis of encephalitis that was present as a comorbidity in this patient. In anti-NMDAR encephalitis, CSF antibodies correlated with outcome and relapse better than serum antibodies, but during long-time follow-ups, both types of antibodies decreased in most patients regardless of the outcomes [41]. Furthermore, in contrast to antibodies against cell-surface antigens, those against intracellular antigens trigger T-cell responses, leading to neuronal death and poorer outcomes [42]. In this setting, circulating antibody titers might not timely reflect the clinical course while remaining instrumental for diagnosis. We plan to continue monitoring antibody titers in this patient and investigate their correlation with severe complications, such as respiratory failure.

ZSCAN1 is a 408 amino acid long protein whose functions remain mostly unknown. It is a transcription factor that belongs to the C2H2 domain-containing zinc finger protein family. In recent years, a number of reports link the high methylation and/or low expression levels of the *ZSCAN1* gene to proliferation in cervical and breast cancers [43,44]. In breast cancer, ZSCAN1 acts as a tumor suppressor by inhibiting TAZ, which is a component of the Hippo (YAP/TAZ) pathway, an intracellular signaling network, the deregulation of which is known to promote tumor growth [45]. Some researchers hypothesize that the Hippo pathway could be involved in the metastasis of neuroblastoma [46]. Furthermore, one study found that ghrelin, a hormone that stimulates appetite, acts through the hypothalamic GHS-R1a receptor to dephosphorylate YAP and control nuclear YAP/TAZ localization and target gene expression [47]. Thus, it might be possible that inactivation through blocking antibodies of ZSCAN1 in the hypothalamus further increases TAZ levels and YAP/TAZ activity, thus enhancing the effect of ghrelin and promoting appetite and, consequently, obesity. Moreover, TAZ is known to increase preadipocyte proliferation [48], and activated YAP/TAZ prevents apoptosis in white adipose cells, thus contributing to obesity [49]. However, the direct implication of ZSCAN1 in regulating the Hippo pathway in the hypothalamus is yet to be demonstrated.

Our study has the limitation of reporting on only one patient with high titers of anti-ZSCAN1 antibodies. Antibodies in the patient’s CSF and tumor were not measured, the follow-up period of this patient has been relatively short, and the titers have not been re-evaluated after a longer period of observation.

## 4. Conclusions and Future Directions

We reported on a patient with a clinical diagnosis of ROHHAD syndrome comorbid with encephalitis, with a rapid progression of symptoms and high titers of anti-ZSCAN1 antibodies, a possible new marker of this syndrome. Based on the course of the patient’s symptoms and the results of immunological studies, CCHS, Prader–Willi syndrome, NMDA encephalitis, etc., were considered less probable. The rapid progression from obesity onset to central hypoventilation and encephalitis within 4 months represents a warning about the severe consequences of ROHHAD syndrome and secondary obesity in general. In addition to raising awareness about this apparently rare and underdiagnosed syndrome, further research is necessary to discern whether anti-ZSCAN1 antibodies are specific to ROHHAD syndrome, whether the antibody titers correlate with the age of symptom onset and prognosis, and whether there is any difference in antibody production between patients with and without tumors.

## Figures and Tables

**Table 1:** Main laboratory data.

	Units	Value	Reference Range
**Venous blood gas (F_I_O_2_ 0.45)**			
pH		7.466	7.350–7.450
pCO_2_	mmHg	35.1	35.0–45.0
**Cell count**			
Hemoglobin	g/dL	10.8	11.6–14.8
White blood cells	/μL	6540	3300–8600
Platelets	×1000/μL	343	158–348
**Electrolytes**			
Sodium	mmol/L	151	138–145
Chloride	mmol/L	115	101–108
Potassium	mmol/L	4.5	3.5–4.9
Calcium	mg/dL	8.7	8.7–10.2
Phosphorus	mg/dL	6.2	4.5–5.8
**Endocrine tests**			
ACTH	pg/mL	9.8	7.2–63.3
Cortisol	μg/dL	4.1	6.0–22.0
17-OHP	ng/mL	0.4	<5.0
DHEA-S	μg/dL	<2.0	<33.2
Prolactin	ng/mL	26.7	1.2–14.4
Growth hormone	ng/mL	0.48	*
IGF-1	ng/mL	11	32–213
TSH	mIU/L	1.84	0.61–4.23
free T4	ng/dL	0.69	1.00–1.80
Renin concentration	pg/mL	101.7	2.5–21.4
Aldosterone	pg/mL	14.9	30–159
Adrenaline (blood)	pg/mL	15	<100
Adrenaline (urine)	μg/mg creatinine	0.006	<39 **
Noradrenaline (blood)	pg/mL	57	<70
Noradrenaline (urine)	μg/mg creatinine	0.052	0.012–0.139 **
Dopamine (blood)	pg/mL	16	<30
Dopamine (urine)	μg/mg creatinine	1.313	0.220–1.654 **
Mean daily urine cortisol	μg/day	28.4	4.3–176.0
**Tumor-related markers**			
NSE	ng/mL	22.8	<15.1
sIL-2R	U/mL	446	156.6–474.5
AFP	ng/mL	<0.9	<6.2
Homovanillic acid (urine)	mg/g creatinine	26.4	7.3–28.6
Vanillylmandelic acid (urine)	mg/g creatinine	15.8	4.8–16.1
**Urine**			
Specific gravity		1.019	1.010–1.025
Proteins		+/−	-
Glucose		-	-
Occult blood		3+	-
Na	mmol/L	157	*
Cl	mmol/L	104	*
K	mmol/L	51.3	*
**Cerebrospinal fluid (CSF)**			
Cell count	/μL	3	<5
Proteins	mg/dL	38	10–40
Glucose	mg/dL	52	50–75
Sodium	mmol/L	160	130–150
Chloride	mmol/L	>120	120–130
Potassium	mmol/L	3.0	2.5–3.5
Oligoclonal bands		Positive	Negative

* No established reference interval for one-time blood/urine tests. ** Reference intervals from [22].

**Table 2:** Values of anti-ZSCAN1 antibodies before and after acute phase treatment.

	Pretreatment	Post- Treatment	Positive Control	Negative Control	Reference
Anti-ZSCAN1 antibodies	348	350	74	3.2	<40

## Data Availability

The data that support the findings of this study are available from the corresponding author upon reasonable request.

## References

[1] Pinhas-Hamiel O., Hamiel U., Bendor C.D., Bardugo A., Twig G., Cukierman-Yaffe T. (2022). The Global Spread of Severe Obesity in Toddlers, Children, and Adolescents: A Systematic Review and Meta-Analysis. Obes. Facts.

[2] Woolford S.J., Sidell M., Li X., Else V., Young D.R., Resnicow K., Koebnick C. (2021). Changes in Body Mass Index among Children and Adolescents During the COVID-19 Pandemic. JAMA.

[3] Fäldt A., Nejat S., Edvinsson Sollander S., Durbeej N., Holmgren A. (2023). Increased Incidence of Overweight and Obesity among Preschool Swedish Children during the COVID-19 Pandemic. Eur. J. Public Health.

[4] Takaya J., Higashino H., Takaya R., Sakaguchi H., Tanoue J., Higashide T., Moriguchi H., Nakao M., Takai Y. (2023). Effect of the COVID-19 Pandemic Lockdown on the Physique of School-Age Children in Japan. Ann. Pediatr. Endocrinol. Metab..

[5] Kass D.A., Duggal P., Cingolani O. (2020). Obesity Could Shift Severe COVID-19 Disease to Younger Ages. Lancet.

[6] Sun S.S., Liang R., Huang T.T.-K., Daniels S.R., Arslanian S., Liu K., Grave G.D., Siervogel R.M. (2008). Childhood Obesity Predicts Adult Metabolic Syndrome: The Fels Longitudinal Study. J. Pediatr..

[7] Zhang T., Whelton P.K., Xi B., Krousel-Wood M., Bazzano L., He J., Chen W., Li S. (2019). Rate of Change in Body Mass Index at Different Ages during Childhood and Adult Obesity Risk. Pediatr. Obes..

[8] Styne D.M., Arslanian S.A., Connor E.L., Farooqi I.S., Murad M.H., Silverstein J.H., Yanovski J.A. (2017). Pediatric Obesity—Assessment, Treatment, and Prevention: An Endocrine Society Clinical Practice Guideline. J. Clin. Endocrinol. Metab..

[9] Ize-Ludlow D., Gray J.A., Sperling M.A., Berry-Kravis E.M., Milunsky J.M., Farooqi I.S., Rand C.M., Weese-Mayer D.E. (2007). Rapid-Onset Obesity with Hypothalamic Dysfunction, Hypoventilation, and Autonomic Dysregulation Presenting in Childhood. Pediatrics.

[10] Fishman L.S., Samson J.H., Sperling D.R. (1965). Primary Alveolar Hypoventilation Syndrome (Ondine’s Curse). Am. J. Dis. Child..

[11] Katz E.S., McGrath S., Marcus C.L. (2000). Late-Onset Central Hypoventilation with Hypothalamic Dysfunction: A Distinct Clinical Syndrome. Pediatr. Pulmonol..

[12] Bougnères P., Pantalone L., Linglart A., Rothenbühler A., Le Stunff C. (2008). Endocrine Manifestations of the Rapid-Onset Obesity with Hypoventilation, Hypothalamic, Autonomic Dysregulation, and Neural Tumor Syndrome in Childhood. J. Clin. Endocrinol. Metab..

[13] Harvengt J., Gernay C., Mastouri M., Farhat N., Lebrethon M., Seghaye M., Bours V. (2020). ROHHAD(NET) Syndrome: Systematic Review of the Clinical Timeline and Recommendations for Diagnosis and Prognosis. J. Clin. Endocrinol. Metab..

[14] Lee J.M., Shin J., Kim S., Gee H., Lee J.S., Cha D.H., Rim J., Park S.-J., Kim J.H., Uçar A. (2018). Rapid-Onset Obesity with Hypoventilation, Hypothalamic, Autonomic Dysregulation, and Neuroendocrine Tumors (ROHHADNET) Syndrome: A Systematic Review. BioMed Res. Int..

[15] Mandel-Brehm C., Benson L., Tran B., Kung A., Mann S., Vazquez S., Retallack H., Sample H., Zorn K., Khan L. (2022). ZSCAN1 Autoantibodies Are Associated with Pediatric Paraneoplastic ROHHAD. Ann. Neurol..

[16] Nakamura-Utsunomiya A. (2022). Autoimmunity Related to Adipsic Hypernatremia and ROHHAD Syndrome. Int. J. Mol. Sci..

[17] Waters P.J., Komorowski L., Woodhall M., Lederer S., Majed M., Fryer J., Mills J., Flanagan E.P., Irani S.R., Kunchok A.C. (2019). A Multicenter Comparison of MOG-IgG Cell-Based Assays. Neurology.

[18] Prüss H., Dalmau J., Harms L., Höltje M., Ahnert-Hilger G., Borowski K., Stoecker W., Wandinger K.P. (2010). Retrospective Analysis of NMDA Receptor Antibodies in Encephalitis of Unknown Origin. Neurology.

[19] Nakamura-Utsunomiya A., Goda S., Hayakawa S., Sonoko S., Hoorn E., Blanchard A., Saito-hakoda A., Kakimoto H., Hachiya R., Kamimura M. (2022). Identification of Clinical Factors Related to Antibody-mediated Immune Response to the Subfornical Organ. Clin. Endocrinol..

[20] Nakamura-Utsunomiya A., Yamaguchi K., Goshima N. (2023). Anti-ZSCAN1 Autoantibodies Are a Feasible Diagnostic Marker for ROHHAD Syndrome Not Associated with a Tumor. Int. J. Mol. Sci..

[21] Isojima T., Kato N., Ito Y., Kanzaki S., Murata M. (2016). Growth Standard Charts for Japanese Children with Mean and Standard Deviation (SD) Values Based on the Year 2000 National Survey. Clin. Pediatr. Endocrinol..

[22] Pussard E., Neveux M., Guigueno N. (2009). Reference Intervals for Urinary Catecholamines and Metabolites from Birth to Adulthood. Clin. Biochem..

[23] Okamatsu C., London W.B., Naranjo A., Hogarty M.D., Gastier-Foster J.M., Look A.T., LaQuaglia M., Maris J.M., Cohn S.L., Matthay K.K. (2009). Clinicopathological Characteristics of Ganglioneuroma and Ganglioneuroblastoma: A Report from the CCG and COG: Ganglioneuroma and Ganglioneuroblastoma. Pediatr. Blood Cancer.

[24] Decarolis B., Simon T., Krug B., Leuschner I., Vokuhl C., Kaatsch P., Von Schweinitz D., Klingebiel T., Mueller I., Schweigerer L. (2016). Treatment and Outcome of Ganglioneuroma and Ganglioneuroblastoma Intermixed. BMC Cancer.

[25] Adam A., Hochholzer L. (1981). Ganglioneuroblastoma of the Posterior Mediastinum: A Clinicopathologic Review of 80 Cases. Cancer.

[26] De Bernardi B., Gambini C., Haupt R., Granata C., Rizzo A., Conte M., Tonini G.P., Bianchi M., Giuliano M., Luksch R. (2008). Retrospective Study of Childhood Ganglioneuroma. J. Clin. Oncol..

[27] Alexander N., Sullivan K., Shaikh F., Irwin M.S. (2018). Characteristics and Management of Ganglioneuroma and Ganglioneuroblastoma-intermixed in Children and Adolescents. Pediatr. Blood Cancer.

[28] Khaytin I., Victor A., Barclay S., Benson L., Slattery S., Rand C., Kurek K., Weese-Mayer D. (2023). Rapid-Onset Obesity with Hypothalamic Dysfunction, Hypoventilation, and Autonomic Dysregulation (ROHHAD): A Collaborative Review of the Current Understanding. Clin. Auton. Res..

[29] Dalmau J., Lancaster E., Martinez-Hernandez E., Rosenfeld M.R., Balice-Gordon R. (2011). Clinical Experience and Laboratory Investigations in Patients with Anti-NMDAR Encephalitis. Lancet Neurol..

[30] Williams P., Wegner E., Ziegler D.S. (2014). Outcomes in Multifocal Neuroblastoma as Part of the Neurocristopathy Syndrome. Pediatrics.

[31] Zaidi S., Gandhi J., Vatsia S., Smith N.L., Khan S.A. (2018). Congenital Central Hypoventilation Syndrome: An Overview of Etiopathogenesis, Associated Pathologies, Clinical Presentation, and Management. Auton. Neurosci..

[32] Barclay S.F., Rand C.M., Borch L.A., Nguyen L., Gray P.A., Gibson W.T., Wilson R.J.A., Gordon P.M.K., Aung Z., Berry-Kravis E.M. (2015). Rapid-Onset Obesity with Hypothalamic Dysfunction, Hypoventilation, and Autonomic Dysregulation (ROHHAD): Exome Sequencing of Trios, Monozygotic Twins and Tumours. Orphanet J. Rare Dis..

[33] Grossi A., Rusmini M., Cusano R., Massidda M., Santamaria G., Napoli F., Angelelli A., Fava D., Uva P., Ceccherini I. (2023). Whole Genome Sequencing in ROHHAD Trios Proved Inconclusive: What’s beyond?. Front. Genet..

[34] Lazea C., Sur L., Florea M. (2021). ROHHAD (Rapid-Onset Obesity with Hypoventilation, Hypothalamic Dysfunction, Autonomic Dysregulation) Syndrome—What Every Pediatrician Should Know About the Etiopathogenesis, Diagnosis and Treatment: A Review. Int. J. Gen. Med..

[35] Victor A.K., Hedgecock T., Donaldson M., Johnson D., Rand C.M., Weese-Mayer D.E., Reiter L.T. (2023). Analysis and Comparisons of Gene Expression Changes in Patient- Derived Neurons from ROHHAD, CCHS, and PWS. Front. Pediatr..

[36] Sartori S., Priante E., Pettenazzo A., Marson P., Suppiej A., Benini F., Perilongo G., Toldo I. (2014). Intrathecal Synthesis of Oligoclonal Bands in Rapid-Onset Obesity with Hypothalamic Dysfunction, Hypoventilation, and Autonomic Dysregulation Syndrome: New Evidence Supporting Immunological Pathogenesis. J. Child Neurol..

[37] Sethi K., Lee Y.-H., Daugherty L.E., Hinkle A., Johnson M.D., Katzman P.J., Sullivan J.S. (2014). ROHHADNET Syndrome Presenting as Major Behavioral Changes in a 5-Year-Old Obese Girl. Pediatrics.

[38] Gharial J., Ganesh A., Curtis C., Pauranik A., Chan J.A., Kurek K., Lafay-Cousin L. (2020). Neuroimaging and Pathology Findings Associated With Rapid Onset Obesity, Hypothalamic Dysfunction, Hypoventilation, and Autonomic Dysregulation (ROHHAD) Syndrome. J. Pediatr. Hematol. Oncol..

[39] Hiyama T.Y., Utsunomiya A.N., Matsumoto M., Fujikawa A., Lin C.-H., Hara K., Kagawa R., Okada S., Kobayashi M., Ishikawa M. (2017). Adipsic Hypernatremia without Hypothalamic Lesions Accompanied by Autoantibodies to Subfornical Organ. Brain Pathol..

[40] Nakamura-Utsunomiya A., Hiyama T.Y., Okada S., Noda M., Kobayashi M. (2017). Characteristic Clinical Features of Adipsic Hypernatremia Patients with Subfornical Organ-Targeting Antibody. Clin. Pediatr. Endocrinol..

[41] Gresa-Arribas N., Titulaer M.J., Torrents A., Aguilar E., McCracken L., Leypoldt F., Gleichman A.J., Balice-Gordon R., Rosenfeld M.R., Lynch D. (2014). Antibody Titres at Diagnosis and during Follow-up of Anti-NMDA Receptor Encephalitis: A Retrospective Study. Lancet Neurol..

[42] Lancaster E., Dalmau J. (2012). Neuronal Autoantigens—Pathogenesis, Associated Disorders and Antibody Testing. Nat. Rev. Neurol..

[43] Boers A., Wang R., van Leeuwen R.W., Klip H.G., de Bock G.H., Hollema H., van Criekinge W., de Meyer T., Denil S., van der Zee A.G.J. (2016). Discovery of New Methylation Markers to Improve Screening for Cervical Intraepithelial Neoplasia Grade 2/3. Clin. Epigenet..

[44] Zhong X., Zhong G. (2021). Prognostic Biomarker Identification and Tumor Classification in Breast Cancer Patients by Methylation and Transcriptome Analysis. FEBS Open Bio.

[45] Chu J., Li Y., He M., Zhang H., Yang L., Yang M., Liu J., Cui C., Hong L., Hu X. (2023). Zinc Finger and SCAN Domain Containing 1, ZSCAN1, Is a Novel Stemness-Related Tumor Suppressor and Transcriptional Repressor in Breast Cancer Targeting TAZ. Front. Oncol..

[46] Cai Y., Chen K., Cheng C., Xu Y., Cheng Q., Xu G., Wu Y., Wu Z. (2020). Prp19 Is an Independent Prognostic Marker and Promotes Neuroblastoma Metastasis by Regulating the Hippo-YAP Signaling Pathway. Front. Oncol..

[47] Zindel D., Mensat P., Vol C., Homayed Z., Charrier-Savournin F., Trinquet E., Banères J.-L., Pin J.-P., Pannequin J., Roux T. (2021). G Protein-Coupled Receptors Can Control the Hippo/YAP Pathway through Gq Signaling. FASEB J..

[48] An Y., Kang Q., Zhao Y., Hu X., Li N. (2013). Lats2 Modulates Adipocyte Proliferation and Differentiation via Hippo Signaling. PLoS ONE.

[49] Wang L., Wang S., Shi Y., Li R., Günther S., Ong Y.T., Potente M., Yuan Z., Liu E., Offermanns S. (2020). YAP and TAZ Protect against White Adipocyte Cell Death during Obesity. Nat. Commun..

